# Identification of Yellow Seed Color Genes Using Bulked Segregant RNA Sequencing in *Brassica juncea* L.

**DOI:** 10.3390/ijms25031573

**Published:** 2024-01-26

**Authors:** Yang Wang, Hong Lu, Xiang Liu, Lu Liu, Wenying Zhang, Zhen Huang, Keqi Li, Aixia Xu

**Affiliations:** State Key Laboratory of Crop Stress Biology for Arid Areas, College of Agronomy, Northwest A&F University, Xianyang 712100, China

**Keywords:** BSR-Seq, *B. juncea*, DEGs, QTL, SNP, yellow seed

## Abstract

Yellow seed breeding is an effective method to improve oil yield and quality in rapeseed (*Brassica napus* L.). However, naturally occurring yellow-seeded genotypes have not been identified in *B. napus.* Mustard (*Brassica juncea* L.) has some natural, yellow-seeded germplasms, yet the molecular mechanism underlying this trait remains unclear. In this study, a BC_9_ population derived from the cross of yellow seed mustard “Wuqi” and brown seed mustard “Wugong” was used to analyze the candidate genes controlling the yellow seed color of *B. juncea*. Subsequently, yellow-seeded (BY) and brown-seeded (BB) bulks were constructed in the BC_9_ population and subjected to bulked segregant RNA sequencing (BSR-Seq). A total of 511 differentially expressed genes (DEGs) were identified between the brown and yellow seed bulks. Enrichment analysis revealed that these DEGs were involved in the phenylpropanoid biosynthetic process and flavonoid biosynthetic process, including key genes such as *4CL*, *C4H*, *LDOX/TT18*, *PAL1*, *PAL2*, *PAL4*, *TT10*, *TT12*, *TT4*, *TT8*, *BAN*, *DFR/TT3*, *F3H/TT6*, *TT19*, and *CHI/TT5.* In addition, 111,540 credible single-nucleotide polymorphisms (SNPs) and 86,319 INDELs were obtained and used for quantitative trait locus (QTL) identification. Subsequently, two significant QTLs on chromosome A09, namely, *qSCA09-3* and *qSCA09-7*, were identified by G’ analysis, and five DEGs (*BjuA09PAL2*, *BjuA09TT5*, *BjuA09TT6*, *BjuA09TT4*, *BjuA09TT3*) involved in the flavonoid pathway were identified as hub genes based on the protein-to-protein network. Among these five genes, only *BjuA09PAL2* and *BjuA09F3H* had SNPs between BY and BB bulks. Interestingly, the majority of SNPs in *BjuA09PAL2* were consistent with the SNPs identified between the high-quality assembled *B. juncea* reference genome “T84-66” (brown-seed) and “AU213” (yellow-seed). Therefore, *BjuA09PAL2*, which encodes phenylalanine lyase, was considered as the candidate gene associated with yellow seed color of *B. juncea*. The identification of a novel gene associated with the yellow seed coloration of *B. juncea* through this study may play a significant role in enhancing yellow seed breeding in rapeseed.

## 1. Introduction

Brassica, a genus of the Brassicaceae family, encompasses significant agricultural and horticultural crops such as *Brassica rapa* L., *Brassica napus* L., and *Brassica juncea* L. [1]. Among these, *B. napus* and *B. juncea* are extensively used for the production of edible vegetable oil, industrial oil, and biofuel, collectively representing the third largest source of vegetable oil globally [2], surpassed only by soybean and sunflower. Therefore, increasing the oil content remains a constant goal of Brassica crop breeding [3]. Studies have shown that yellow-seeded rapeseed has higher oil and protein contents and lower crude fiber content compared to brown- or black-seeded rapeseed [4].

Although a large number of yellow-seeded varieties of *B. napus* have been developed through interspecific crosses [5]. Natural occurrences of yellow-seeded genotypes have not been identified in *B. napus* [6]. Due to its commercial importance, researchers have been studying the mechanism of yellow seed trait over the past decades. To elucidate the pigment compounds associated with this specific trait, a number of studies have been conducted. The manifestation of yellow seed color in rapeseed is largely determined by the presence of phenolic compounds [7,8,9]. The main phenolic compounds found in rapeseed are flavonoids [10]. Marles [11] found that the main pigments that affect the seed color are proanthocyanins (PAs). PAs are the end-products of the flavonoid biosynthesis pathway [12,13], a pathway that has been extensively explored at the genetic and biochemical levels in model plants such as *Arabidopsis thaliana* and *Zea mays* [14,15] Furthermore, certain studies have indicated that melanin exerts a significant effect on seed coat color [16]. In *B. napus*, seed color is controlled by different candidate genes depending on the genetic background, and the QTLs controlling seed color have been identified using classical genetic tools in diverse population [17,18,19,20,21,22]. However, most studies focus on *B. napus*, and the inheritance of yellow seed trait in *B. napus* is complex.

On the other hand, *B. juncea*, an allotetraploid species, harbors naturally occurring yellow-seeded germplasms with stable inheritance. Cultivated globally in countries such as India, China, Bangladesh, Ukraine, Canada, and Australia due to its wide adaption [23], *B. juncea* can be categorized into three classes, yellow-seeded, black-seeded, and brown-seeded. Therefore, cultivating high-yield and superior-quality yellow-seeded *B. juncea* is considered as one of the most important objectives for rapeseed breeding. Notably, the yellow-seeded landrace “Wuqi” mustard, a variety of *B. juncea*, has been cultivated in Northern Shaanxi, China. The yellow seed trait is controlled by a single recessive gene [24], offering a potential avenue for transferring the yellow seed gene to develop yellow-seeded *B. napus*. Despite this potential, few studies have been conducted on the mechanistic aspects of yellow seed development in *B. juncea*. Therefore, understanding the mechanism controlling the yellow seed trait in “Wuqi” is important for the oilseed industry. Previous studies have identified the A09 chromosome as the location of yellow seed color gene in “Wuqi” mustard, and a high-resolution genetic and physical map around this gene has been constructed [24]. However, the mechanism of yellow seed coat color remains elusive, and no gene controlling yellow seed coat color has been isolated to date. Some studies have indicated that the yellow seed color genes in *Brassica* are linked to the flavonoid biosynthesis pathway [25]. Whether the candidate genes identified in our study also participate in the flavonoid biosynthesis pathway is yet to be understood. Therefore, the objective of this study is to identify the candidate genes responsible for yellow seed color in *B. juncea*. The outcomes of this investigation will lay a strong foundation for comprehending the mechanism of yellow seed color in *Brassica* crops.

## 2. Result

### 2.1. Critical Period of Seed Color Formation

**Figure 1:**
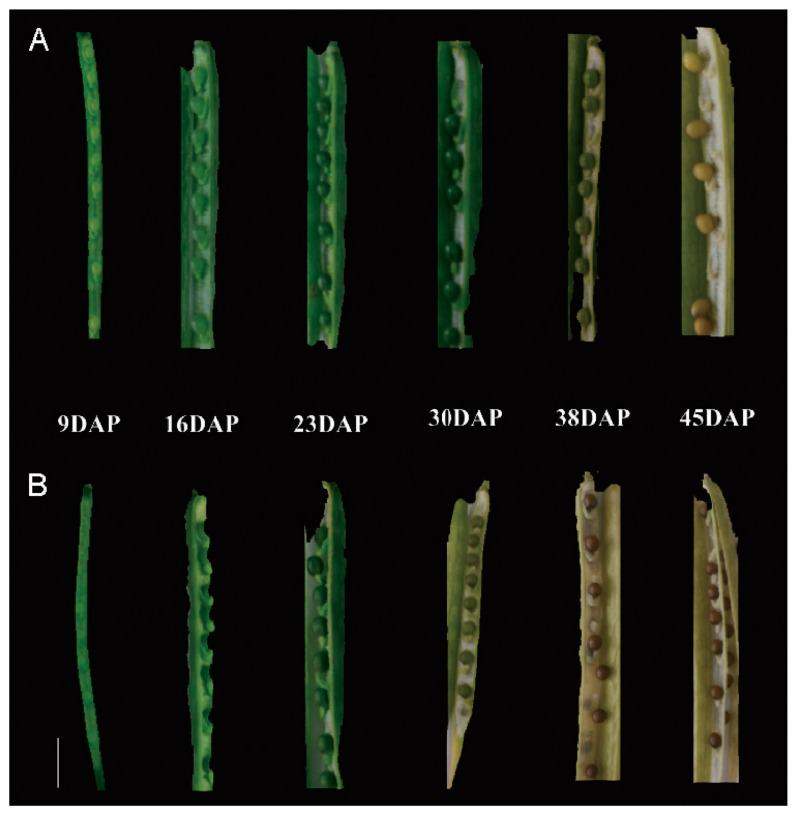
Seed color changes during seed development. Seed color changes during seed development in yellow-seeded “Wuqi” mustard (**A**, scale bar = 1 cm) and brown-seeded “Wugong” mustard (**B**, scale bar = 1 cm).

To investigate the seed color formation process, we compared the seed colors of “Wuqi” and “Wugong” mustards at various seed development stages (Figure 1). In “Wuqi” mustard, the seed color exhibited a progression from light green at 9 to 16 days after pollination (DAP) to dark green at 23 and 30 DAP, followed by a transition to yellow at 38 DAP and eventually to full yellow at 45 DAP. In contrast, the seed of “Wugong” mustard displayed a light green color at 9 to 16 DAP, transitioning to dark green at 23 DAP and brown color at 30 DAP. The brown coloration became more pronounced at 38 and 45 DAP. These observations indicate a noticeable divergence in seed coat color between “Wuqi” and “Wugong” mustards, which becomes evident from 30 DAP. 

The levels of flavonoids, anthocyanins, melanin, and total phenol were assessed at different seed developmental stages of “Wuqi” and “Wugong” mustards. The anthocyanin content and total phenol were consistently high at all stages, with the “Wugong” mustard exhibiting higher concentrations compared to the “Wuqi” mustard. During the developmental stages of seeds, the contents of flavonoid (except at 9 DAP and 16 DAP), anthocyanidin, and melanin and the total phenol of the “Wugong” mustard were significantly higher than those in the “Wuqi” mustard (*p* < 0.01). The contents of flavonoids, melanin, and total phenol in both “Wuqi” mustard and “Wugong” mustard steadily increased during seed development, with the maximum value detected at 45 DAP. Notably, the synthesis of these compounds occurred earlier in the “Wugong” mustard than the “Wuqi” mustard. The difference in flavonoid and anthocyanin contents in the two parents reached its maximum at 38 DAP and 30 DAP, respectively (Table 1). Therefore, the critical period in seed color formation was identified as 30 DAP.

### 2.2. Alignment of BSR-Seq

In order to investigate the molecular mechanisms underlying the difference in seed coat colors between yellow-seeded and brown-seeded *B. juncea* and to pinpoint key genes regulating this trait, 30 extreme yellow-seeded (BY) and 30 brown-seeded (BB) samples at 30 DAP were selected from the BC_9_ population, derived from the cross between “Wuqi” and “Wugong” mustards, for bulked segregant RNA sequencing (BSR-Seq). 

**Figure 2:**
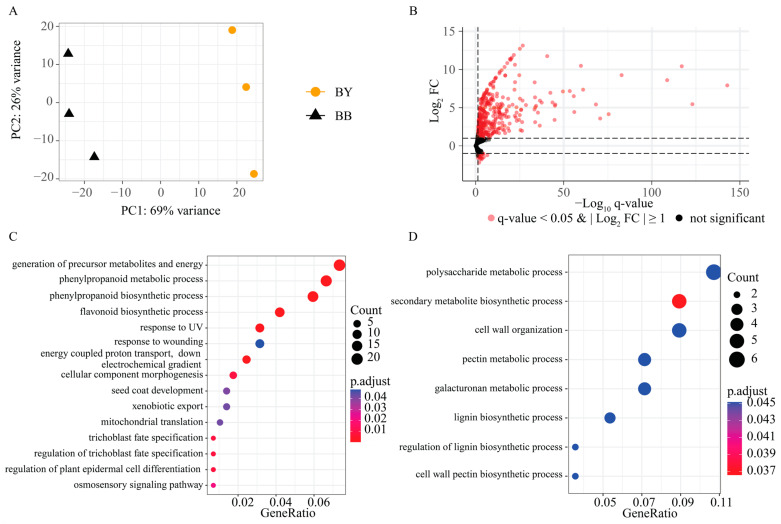
Transcriptome analysis of the BB and BY bulks. (**A**) Principal component analysis (PCA) of transcriptomic data. Different colored dots represent the BB bulks (black) and BY bulks (yellow). (**B**) The volcano plot compares gene expression between these two bulks. Negative log_10_ *p*-values from the differential expression test were plotted against the log_2_ fold change (BB/BY) for each gene. Each dot represents a gene. (**C**) Enriched GO terms of DEGs exhibiting higher expression in BB compared to BY. (**D**) Enriched GO terms of DEGs exhibiting lower expression in BB compared to BY.

A total of 79.3 million clean reads were generated from the BB and BY pooled samples through BSR-Seq. After removing adaptor sequences and low-quality reads, RNA-seq generated 20,397,430–25,216,875 and 23,597,033–26,416,796 clean read pairs for the three replicates of BB and BY bulks, respectively. The percentage of bases with a quality score of Q30 exceeded 90.00%. Furthermore, 83.14–86.06% of the reads for the BB bulks aligned to those of the *B. juncea* cv.AU213 V1.0 [26] reference genome, in comparison to 89.19–90.11% of the reads for the BY bulks (Supplementary Table S1). Principal component analysis (PCA) revealed a clustering of replicates from each bulk along PC1 and PC2, collectively explaining 95% of the variance between samples (Figure 2A). These results affirm the accuracy and high quality of the sequencing data for BSR-Seq analysis, validating its suitability for further analysis.

### 2.3. Identification of DEGs between the BY and BB Bulks

RNA-Seq analysis was performed to identify the DEGs between the BY and BB bulks. Of the 56,260 identified expressed genes, 511 were recognized as DEGs, meeting the criteria of |log2 FC| > 1 and padj < 0.05. Among these genes, 438 and 73 DEGs were up-regulated and down-regulated in BB bulks compared to BY bulks, respectively (Figure 2B and Supplementary Table S2). Furthermore, the up-regulated DEGs were predominantly enriched in biological processes such as phenylpropanoid metabolic process (GO:0009698), flavonoid biosynthetic process (GO:0009813), generation of precursor metabolites and energy (GO:0006091), and response to UV (GO:0009411) (Figure 2C). The down-regulated DEGs were mainly enriched in the pectin metabolic process (GO:0045488) (Figure 2D).

### 2.4. QTL Mapping of Seed Color Using BSR-Seq

**Figure 3:**
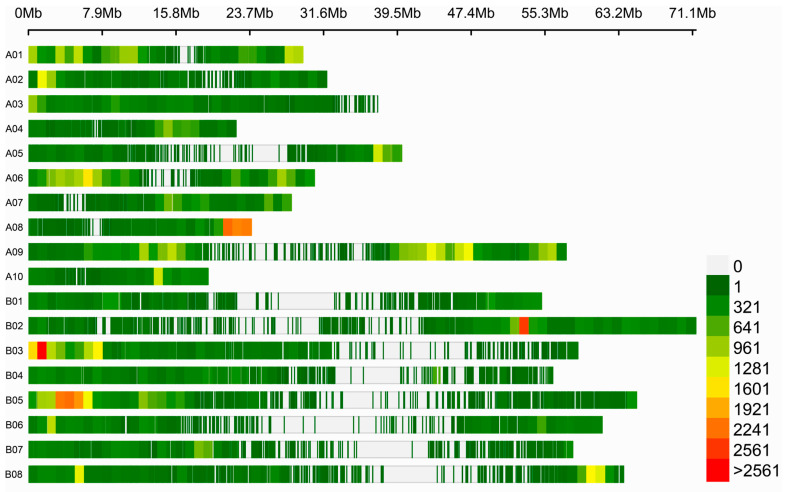
Distribution of SNPs/INDELs on 18 chromosomes. The marker density is indicated by different bar colors, and each bar represents 1 Mb window size.

To identify candidate QTLs related to yellow seed color, a total of 642,380 polymorphic SNPs and 388,259 INDELs between the pooled BB and BY samples were identified. After removing low read depth and low-quality SNPs and INDELs, 111,540 SNPs and 86,319 INDELs were retained for subsequent G’ analysis. Of these SNPs, 61,348 (55%) and 50,192 (45%) were situated on the A and B sub-genomes, respectively. Among these SNPs, 96,022 (76.91%) were located in exon regions, 9954 (7.973%) in intergenic regions, 7230 (5.791%) in intron regions, 6848 (5.49%) in 3′-UTR, and 3589 (2.88%) in 5′-UTR. Regarding the INDELs, 41,904 (49%) and 44,415 (51%) were distributed on the A and B sub-genomes, respectively (Figure 3 and Supplementary Table S3). The numbers of informative SNPs across 18 chromosomes ranged from 2276 (A10) to 15,545 (A09), while the numbers of informative INDELs ranged from 2,16 (A04) to 6974 (A09).

**Figure 4:**
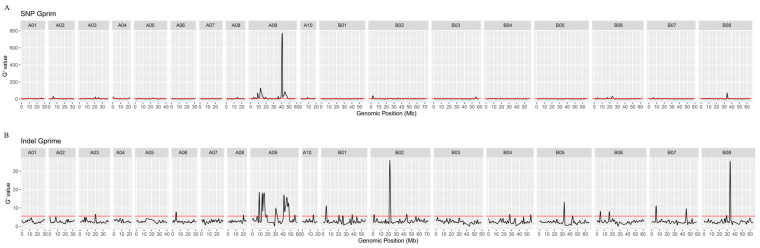
Quantitative trait loci (QTL) for yellow seed color identified using QTLseqr. Plots produced by the *plotQTLStats* function with a 1 Mb sliding window. The tricube-smoothed G’ value for SNP (**A**), and the tricube-smoothed G’ value for INDEL (**B**). The genome-wide false discovery rate threshold of 0.05 is indicated by the red line.

Subsequently, G’ analysis was used for mapping seed coat color genes. Seven significant QTLs were mapped on chromosome A09 with G’ analysis for SNPs. Among these, QTL *qSCA09-7*, spanning from 38.39 to 48.33 Mb, exhibited the highest mean G’ value, while QTL *qSCA09-3*, covering 8.26 to 17.32 Mb, emerged as the second most significant (Figure 4). The G’ values for the QTLs on other chromosomes were only slightly higher than the threshold, indicating that these were minor QTLs (Supplementary Table S4). It is worth noting that the regions of QTL *qSCA09-3* and *qSCA09-7* were also identified with G’ analysis for INDELs (Figure 4, Supplementary Table S5). Consequently, QTL *qSCA09-3* and *qSCA09-7* on chromosome A09 were identified as candidate QTLs associated with seed coat color. This region encompasses a total of 2724 annotated genes, as per the genome annotation information.

### 2.5. Overlapping Candidate Intervals and DEGs

**Figure 5:**
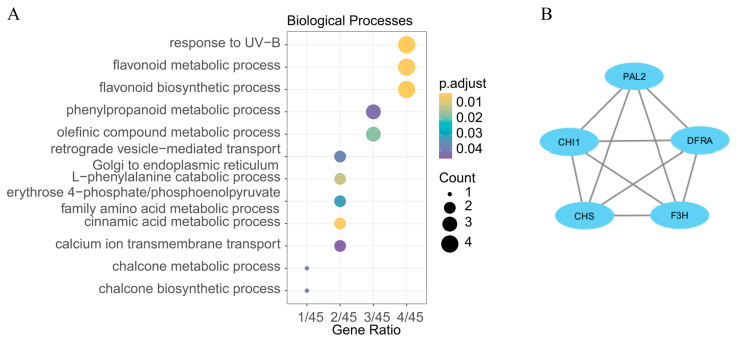
Functional analysis of the candidate genes. (**A**) GO enrichment of DEGs in the region of *qSCA09-3* and *qSCA09-7* on chromosome A09. (**B**) PPI network for the hub genes involved in seed coloration of *B. juncea*.

In order to comprehensively and accurately identify genes governing seed coat color, 51 DEGs located in the region of *qSCA09-3* and *qSCA09-7* were identified as potential candidates. These candidate genes exhibited enrichment in GO terms, including response to UV-B and flavonoid biosynthetic process (Figure 5A and Table 2). To explore the molecular mechanism of seed color formation in *B. juncea*, protein–protein interaction (PPI) networks were constructed using the STRING database with these 51 candidate genes. Strikingly, the PPI networks of the candidate genes highlighted a predominant association with the flavonoid biosynthesis pathways (Figure 5B). Among these genes, *BjuOA09G47290* (*BjuA09PAL2*), *BjuOA09G49050* (*BjuA09CHI1*), *BjuOA09G41640* (*BjuA09CHS*), *BjuOA09G45170* (*BjuA09F3H*), and *BjuOA09G20700* (*BjuA09DFRA*) emerged as the hub genes in the network, suggesting they might play critical roles in the seed color formation of *B. juncea*. 

However, only two genes, namely, *BjuA09PAL2* and *BjuA09F3H*, exhibited polymorphism between the BB and BY bulks (Table 3). We further compared the number of SNPs between the BY and BB bulks in these two genes. The analysis revealed 30 and 6 SNPs in *BjuA09PAL2* and *BjuA09F3H*, respectively. To elucidate the role of these SNPs in the formation of yellow seed color, we compared the sequence of *BjuA09PAL2* and *BjuA09F3H* in two additional *B. juncea* accessions: “T84-66” (brown-seed) [26] and “AU213” (yellow-seed) [26], respectively. Just one SNP was identified in *BjuA09F3H* between “T84-66” (brown-seed) and “AU213” (yellow-seed), and notably, this SNP did not align with the SNPs between BY and BB bulks. However, 23 SNPs between “T84-66” and “AU213” were identified in the region of *BjuA09PAL2*. Interestingly, 20 of these SNPs aligned with the SNPs between BY and BB bulks (Table 3). These results suggested that *BjuA09PAL2* may be a pivotal gene associated with the regulation of seed color. Importantly, the *Arabidopsis* homolog of *BjuA09PAL2* is *AtPAL2*, which encodes phenylalanine lyase.

## 3. Discussion

Yellow seed has been considered as a desirable trait related to seed quality for rapeseed breeders. However, the predominant approach for developing all-yellow seed materials in *B. napus* involves interspecific hybridization, resulting in challenges such as a low yellow seed rate and seediness [27,28]. Therefore, it is essential to explore the molecular mechanism of the yellow seed coloration of *B. juncea*, which possesses natural and original yellow seed genetic resources. In this study, we used a BC_9_ population derived from the cross of yellow-seeded mustard “Wuqi” and brown-seeded mustard “Wugong” to identify candidate genes controlling yellow seed color in *B. juncea*. 

Over the years, substantial progress has been made in understanding the physiological and biochemical mechanisms of yellow seed coloration in rapeseed. In this study, we found that the products of the flavonoid pathway, such as flavonoid and anthocyanin, exhibited higher accumulation in brown-seeded mustard compared to yellow-seeded mustard, which is in agreement with the previous findings that the difference in seed coat color between the black seed and yellow seed is closely linked to polyphenols, flavonoids, anthocyanins, and melanin in *Brassica* species, maize, and wheat [16,29,30]. Compared with yellow-seeded mustard, the expression of most genes involved in the flavonoid synthesis pathway was significantly up-regulated in brown-seeded mustard, including *4CL*, *C4H*, *LDOX/TT18*, *PAL1*, *PAL2*, *PAL4*, *TT10*, *TT12*, *TT4*, *TT8*, *BAN*, *DFR/TT3*, *F3H/TT6*, *TT19*, and *CHI/TT5.* The expression difference of genes involved in the flavonoid pathway was consistent with the previous findings [31,32,33]. Therefore, the mechanism of yellow seed coat coloration of *B. juncea* can be elucidated based on the variations in these key biochemical indices. 

To date, numerous QTLs [19,21,34,35,36] and candidate genes [37,38,39,40] have been reported to be involved in determining the seed color of *B. napus* through methods such as QTL mapping, comparative genomic analysis, resequencing analysis, transcriptome analysis, and metabolome analysis. With the development of next-generation sequencing technology and the decline in sequencing costs, the genome sequence of many essential species has been successfully obtained. BSR-Seq, an economical and effective approach for gene mapping, is particularly valuable for species with reference genomes. Currently, the BSR-Seq mapping strategy has been widely used to map key genes in *Brassica*, maize, wheat, and other species [41,42,43,44]. In contrast to traditional methods, BSR-Seq yields comprehensive genetic information, such as SNPs and gene expression data, which greatly accelerates the process of gene mapping. In this study, BSR-Seq was used to locate yellow seed color genes on the A09 chromosome, which is consistent with previous studies which uncovered that the seed color of *B. napus* is regulated by a limited number of QTLs located on chromosome A09 [20,21,35]. 

Several genes have been functionally validated in *B. napus*, such as *TT7*, *TT18*, *TT10*, *TT1*, *TT2*, and *TT12* [5,38,39]. The majority of genes identified in Brassica species as regulators of yellow seed color are associated with the flavonoid synthesis pathway and anthocyanin synthesis pathway. In this study, we identified a novel candidate gene, BjuPAL2, potentially controlling the yellow seed color trait by integrating DEG analysis, BSR analysis, and comparative genomic analyses of published yellow- and brown-seeded reference genomes. Phenylalanine ammonia-lyase (PAL) initiates the phenylpropanoid pathway, which produces a range of important secondary metabolites. Serving as the initial phase of the flavonoid synthesis pathway, the phenylpropanid synthesis pathway plays an important role in anthocyanin synthesis. There are four *PAL* genes (*PAL1*, *PAL2*, *PAL3*, and *PAL4*) in Arabidopsis. Huang et al. reported that the double mutants of *PAL1* and *PAL2* produced yellow seeds and attributed this to the absence of condensed tannin pigments in the seed. These mutants exhibited a heightened sensitivity to ultraviolet-B light but an increased tolerance to drought [45]. Consequently, exploring the additional functions of *PAL* in Brassica species is imperative for a comprehensive understanding of the role of *PAL* in plants. Further studies are needed to verity its function in rapeseed using transgenic technology.

## 4. Materials and Methods

### 4.1. Plant Materials and Growth Condition

Inbred lines of “Wuqi” (yellow-seeded) and “Wugong” (brown-seeded) mustard, along with the backcross 9 population (BC9) derived from the cross between “Wuqi” and “Wugong” (“Wuqi” is the recurrent parent) were used in this study. The parental lines were cultivated in a greenhouse (Northwest A&F University, Yangling, Shaanxi, China), maintaining a temperature of 25 °C during the day (16 h) and 20 °C at night (8 h). Seeds at various developmental stages (9, 16, 23, 30, 38, and 45 DAP) from both parental lines were systematically observed and documented. Meanwhile, these seeds were collected, immediately frozen in liquid nitrogen and stored at −80 °C. Plants of BC_9_ were grown in the field station of Northwest A&F University for BSR-Seq. 

### 4.2. Determination of Total Flavonoids, Anthocyanin, Total Phenol, and Melanin Content

The contents of flavonoids, anthocyanin, and total phenol were measured by a colorimetric assay with a hydrochloric acid methanol method [46]. A total of 0.1 g seed was ground in 5 mL acetone (Sigma-Aldrich, Shanghai, China), and then incubated in water bath at 30 °C for 1 h to extract chlorophyll and lutein. Subsequently, the mixture was centrifuged at 3500 r/min for 15 min, and the supernatant was discarded. The precipitate was re-suspended in 5 mL methanol containing 5% hydrochloric acid and incubated in water bath at 60 °C for 1 h. The supernatant was collected, and the precipitate was re-extracted three times following the forementioned procedure. The extracts were combined and diluted in methanol containing 5% hydrochloric acid to a total volume of 10 mL. The content of total phenol [46], flavonoid [47], and anthocyanin [46] was determined by measuring the absorbance using spectrophotometer at 280 nm, 325 nm, and 530 nm, respectively. Regarding melanin, the measurement process is basically the same, except for the last step. After incubation at 60 °C for 1 h, the precipitate was immersed in 2 mL 2% NaOH, and then incubated in a 70 °C water bath until the color of the material completely faded. The melanin content was determined by measuring the absorbance of the mixture at 290 nm using spectrophotometer.

### 4.3. Bulk Construction and Bulked Segregant RNA Sequencing 

At 30 DAP, seeds from individual plants in the BC_9_ population were collected and stored in liquid nitrogen. The seed color of each individual plant was recorded when the seeds were mature, and the seeds were categorized into yellow-seed (BY) and brown-seed (BY) bulks. Prior to RNA extraction, the seeds were pooled, with each pool consisting of 30 seeds from lines of BY and BB bulks, respectively. The number of BC_9_ lines in each bulk is listed in Table S1, and each bulk comprises 3 biological replicates for RNA sequencing (BSR-Seq). An RNA prep Pure Plant Kit (TIAN GEN, Beijing, China) was used for RNA extraction from each sample following the manufacturer’s instructions. The RNA concentration and quality were checked using a NanoDrop 2000c Spectrophotometer and an Agilent Bioanalyzer (RIN) for each sample. The mRNA was isolated and concentrated using magnetic beads attached with oligo d(T) for cDNA library preparation. The first-strand cDNA was synthesized from the mRNA using random hexamers. The cDNA libraries were prepared by ligating the cDNA fragments to the Illumina adapter followed by PCR amplification and purification with AMPure XP beads. The libraries were sequenced on an Illumina HiSeq 2000 platform with paired-end 150 bp reads by the SAGENE Company in Guangzhou, China. 

### 4.4. Differentially Expressed Gene (DEG) Analysis

The raw reads with 150 paired-end base pair (bp) were filtered, and the clean reads were mapped to the *Brassica juncea* cv.AU213 V1.0 reference genome using Hisat2. Gene read counts were quantified by HTSeq. To filter genes with very low expression, a read count threshold of 5 was applied, retaining only those genes with at least 5 reads in one sample for subsequent analysis. The DESeq2 R package was employed to conduct differential expression analysis between the BY and BB pools. Genes were identified as differentially expressed genes if they met the criteria of |log2 FC| ≥ 1 and adjusted *p*-value (FDR) < 0.01. A principal component analysis (PCA) was carried out and visualized by the plotPCA() function in the DESeq2 package. We used homology-based methods to characterize the function of the DEGs in *B. juncea*, and the protein sequences of DEGs were aligned to *Arabidopsis* protein sequences using BLASTP (an E value cutoff of 1× 10^−10^). Gene Ontology (GO) enrichment analysis was performed by the clusterProfiler R package. GO terms with a corrected FDR ≤ 0.05 were considered significantly enriched. 

### 4.5. Variant Detection and BSA (Bulk Segregant Analysis) Association Mapping

Prior to conducting the bulk segregant analysis, the sequencing reads from each individual were mapped to the *Brassica juncea* cv.AU213 V1.0 reference genome using STAR with two pass-mode. Additionally, subsequent SNP calling was identified by GATK 4.2.2.0. The variant information of each replicate was merged by the GATK for BY and BB bulks. SNPs and INDEls were first filtered for lower mapping quality (MQ < 25) and lower sequencing depth (DP < 3) and reference allele frequency < 0.05. After filtering, 111,540 SNPs and 86,319 INDELs were used for further analysis. 

We performed BSA using G’ statistical approaches implemented in the QTLseqr [48] R package. Using the smoothed G’ statistic enables noise reduction and effectively addresses linkage disequilibrium between SNPs. The tricube smoothed Δ (SNP-index) and Δ (INDEL-index) were calculated within a 1 Mbp sliding window for both BB and BY bulks. The G and G’ statistics were calculated based on observed and expected allele depths, with smoothing achieved using a tricube smoothing kernel [49]. *p*-values were estimated using the non-parametric method described by Magwene [49]. The genomic region responsible for seed color was defined based on a significance threshold of Benjamini–Hochberg adjusted *p*-value < 0.05.

## 5. Conclusions

Overall, based on the BSR-Seq analysis, two candidate QTLs related to yellow trait in *B. juncea* were located on chromosome A09. In addition, DEG analysis showed that a large proportion of DEGs were enriched to flavonoid biosynthetic process. Meanwhile, five DEGs involved in the flavonoid biosynthetic process (*BjuA09PAL2*, *BjuA09TT5*, *BjuA09TT6*, *BjuA09TT4*, *BjuA09TT3*) were located in the two candidate QTLs. Combining comparative genomic analyses of published yellow- and brown-seeded reference genomes, we identified a novel candidate gene, *BjuA09PAL2*, potentially controlling the yellow seed trait of *B. juncea*. The candidate gene enables the development of molecular markers and will provide support for yellow seed breeding in rapeseed using molecular marker assisted selection.

## Figures and Tables

**Table 1:** Analysis of seed color-related indexes during the development stages of seeds.

	Flavonoids (OD Unit 325 nm/g DW)			Anthocyanins (OD Unit 530 nm/g DW)			Melanin (OD Unit 290 nm/g DW)			Total Phenolics (mg/g DW)		
	Wugong	Wuqi	Difference	Wugong	Wuqi	Difference	Wugong	Wuqi	Difference	Wugong	Wuqi	Difference
9 DAP	121.47	127.85	−6.38	238.20	69.17	169.03 **	26.68	17.60	9.07 **	230.20	120.60	109.60 **
16 DAP	109.51	126.12	−16.61	726.75	75.33	651.42 **	59.89	48.22	11.66 **	319.30	180.90	138.40 **
23 DAP	523.20	337.39	185.81 **	2217.00	182.80	2034.20 **	109.66	87.50	22.15 **	793.30	255.60	537.70 **
30 DAP	887.38	770.32	117.07 **	2337.33	247.83	2089.50 **	224.68	136.51	88.16 **	1070.40	544.00	526.40 **
38 DAP	1926.05	1400.00	526.05 **	1256.34	305.43	950.91 **	397.66	195.95	201.71 **	1157.40	655.50	501.90 **
45 DAP	2035.07	1778.57	256.50 **	1195.00	300.08	894.92 **	698.76	203.55	495.21 **	1229.70	868.00	601.70 **

** indicates significance at the level of 1% determined with *t*-test.

**Table 2:** Location of the candidate DEGs in the QTLs.

QTL	Chrom	Start	End	FPKM in BB	FPKM in BY	Gene ID	At_ID
*qSCA09-3*	A09	8,929,403	8,931,985	914.09	33.40	*BjuOA09G16530*	*AT1G62750*
*qSCA09-3*	A09	9,495,191	9,500,364	14.39	1.43	*BjuOA09G17120*	*AT1G62120*
*qSCA09-3*	A09	11,771,003	11,772,142	182.41	19.20	*BjuOA09G20180*	*AT2G03750*
*qSCA09-3*	A09	11,913,894	11,916,103	224.83	36.06	*BjuOA09G20410*	*AT5G42370*
*qSCA09-3*	A09	13,787,173	13,788,148	54.53	125.96	*BjuOA09G22750*	*AT1G26470*
*qSCA09-3*	A09	14,094,583	14,098,369	51.18	7.05	*BjuOA09G23160*	*AT5G46690*
*qSCA09-3*	A09	14,113,829	14,115,475	307.67	145.43	*BjuOA09G23180*	*AT5G46700*
*qSCA09-3*	A09	14,279,969	14,280,275	283.29	103.14	*BjuOA09G23340*	*AT5G46871*
*qSCA09-3*	A09	14,415,615	14,416,772	98.94	288.38	*BjuOA09G23480*	*AT5G47060*
*qSCA09-3*	A09	14,667,647	14,669,807	67.84	165.38	*BjuOA09G23850*	*AT5G47650*
*qSCA09-3*	A09	15,097,228	15,100,123	13.20	0.00	*BjuOA09G24380*	*AT1G62200*
*qSCA09-3*	A09	16,516,247	16,518,639	28.12	1.67	*BjuOA09G26270*	*AT4G04710*
*qSCA09-3*	A09	16,848,640	16,849,093	52.31	110.67	*BjuOA09G26840*	*AT1G64590*
*qSCA09-3*	A09	16,951,516	16,952,504	44.29	108.10	*BjuOA09G27000*	*AT4G12580*
*qSCA09-3*	A09	12,155,595	12,157,288	5727.33	3.97	*BjuOA09G20700*	*AT5G42800*
*qSCA09-3*	A09	12,694,439	12,698,035	44.80	1.13	*BjuOA09G21470*	*AT1G51540*
*qSCA09-3*	A09	13,580,386	13,583,345	36.50	87.50	*BjuOA09G22510*	*AT1G52360*
*qSCA09-7*	A09	38,731,200	38,733,688	158.05	0.26	*BjuOA09G35390*	*At1G32150*
*qSCA09-7*	A09	38,797,903	38,800,210	42.41	10.28	*BjuOA09G35460*	*AT1G32080*
*qSCA09-7*	A09	38,972,655	38,973,252	40.24	90.11	*BjuOA09G35740*	*AT2G37420*
*qSCA09-7*	A09	39,070,816	39,072,813	1244.90	551.60	*BjuOA09G35800*	*AT1G31830*
*qSCA09-7*	A09	39,216,208	39,218,452	54.76	117.49	*BjuOA09G35960*	*AT1G31660*
*qSCA09-7*	A09	40,466,891	40,467,856	14.78	51.11	*BjuOA09G37390*	*AT5G26770*
*qSCA09-7*	A09	40,486,136	40,487,627	58.47	139.95	*BjuOA09G37430*	*AT1G73490*
*qSCA09-7*	A09	40,724,953	40,725,511	33.64	82.22	*BjuOA09G37660*	*AT1G33390*
*qSCA09-7*	A09	42,041,441	42,044,539	60.86	0.00	*BjuOA09G39430*	*AT1G28020*
*qSCA09-7*	A09	42,288,652	42,290,857	18.15	0.00	*BjuOA09G39810*	*AT1G27500*
*qSCA09-7*	A09	42,429,480	42,430,621	10.62	50.20	*BjuOA09G40150*	*AT1G63660*
*qSCA09-7*	A09	42,537,814	42,540,009	27.12	93.94	*BjuOA09G40310*	*AT5G18880*
*qSCA09-7*	A09	42,765,028	42,767,784	249.60	74.33	*BjuOA09G40600*	*AT1G25390*
*qSCA09-7*	A09	42,796,085	42,797,338	52.17	2.46	*BjuOA09G40630*	*AT3G20620*
*qSCA09-7*	A09	42,873,467	42,875,000	29.26	1.41	*BjuOA09G40750*	*AT1G14800*
*qSCA09-7*	A09	42,894,662	42,895,078	18.93	0.85	*BjuOA09G40790*	*AT1G21380*
*qSCA09-7*	A09	42,895,126	42,896,198	12.29	0.28	*BjuOA09G40800*	*AT1G14800*
*qSCA09-7*	A09	42,915,568	42,917,540	78.09	32.19	*BjuOA09G40860*	*AT1G25520*
*qSCA09-7*	A09	43,276,364	43,278,604	34.41	3.97	*BjuOA09G41440*	*AT1G26680*
*qSCA09-7*	A09	43,368,606	43,369,235	26.12	1.14	*BjuOA09G41640*	*AT5G13930*
*qSCA09-7*	A09	43,401,376	43,402,160	69.82	12.92	*BjuOA09G41660*	*AT1G26920*
*qSCA09-7*	A09	44,111,932	44,114,261	184.46	646.93	*BjuOA09G42790*	*AT1G23200*
*qSCA09-7*	A09	44,403,018	44,404,800	16.43	0.86	*BjuOA09G43350*	*AT1G22620*
*qSCA09-7*	A09	44,436,051	44,436,732	29.02	69.92	*BjuOA09G43370*	*AT1G22590*
*qSCA09-7*	A09	44,589,631	44,591,503	81.63	18.31	*BjuOA09G43580*	*AT1G22280*
*qSCA09-7*	A09	45,670,071	45,671,807	2727.30	475.40	*BjuOA09G45170*	*AT3G51240*
*qSCA09-7*	A09	45,707,274	45,708,740	520.50	1235.15	*BjuOA09G45260*	*AT3G51300*
*qSCA09-7*	A09	46,016,674	46,017,162	20.52	1.07	*BjuOA09G45830*	*AT1G79990*
*qSCA09-7*	A09	46,019,207	46,019,883	200.76	46.18	*BjuOA09G45840*	*AT5G38980*
*qSCA09-7*	A09	46,109,849	46,111,124	38.86	7.12	*BjuOA09G45990*	*AT3G51930*
*qSCA09-7*	A09	46,507,649	46,510,728	24.76	65.24	*BjuOA09G46830*	*AT1G72880*
*qSCA09-7*	A09	46,669,636	46,671,877	90.67	193.86	*BjuOA09G47060*	*AT2G23790*
*qSCA09-7*	A09	46,798,481	46,801,460	2511.67	870.15	*BjuOA09G47290*	*AT3G53260*
*qSCA09-7*	A09	47,714,123	47,726,747	37.96	0.28	*BjuOA09G49050*	*AT3G55120*

**Table 3:** Comparison of SNPs in BY and BB and SNPs in “AU213” and “T84-66” (V2).

SNP between BB and BB Bulks					SNP between “AU213” and “T84-66” (V2)			
CHROM	Position	Ref	Alt	Annotation	AU213 Position	Base in AU213	Base in T84-66	Gene ID
A09	46,798,684	T	C	3_prime_UTR_variant	46,798,684	T	C	*BjuOA09G47290*
A09	46,799,098	G	A	synonymous_variant	46,799,098	G	A	*BjuOA09G47290*
A09	46,799,171	T	C	missense_variant	46,799,171	T	C	*BjuOA09G47290*
A09	46,799,228	G	C	missense_variant	46,799,228	G	C	*BjuOA09G47290*
A09	46,799,260	G	A	synonymous_variant	46,799,260	G	A	*BjuOA09G47290*
A09	46,799,300	T	C	missense_variant	46,799,300	T	C	*BjuOA09G47290*
A09	46,799,477	A	T	missense_variant	46,799,477	A	T	*BjuOA09G47290*
A09	46,799,528	A	C	missense_variant	-	-	-	*BjuOA09G47290*
A09	46,799,543	A	G	missense_variant	-	-	-	*BjuOA09G47290*
A09	46,799,606	A	G	missense_variant	46,799,606	A	G	*BjuOA09G47290*
A09	46,799,690	G	C	missense_variant	46,799,690	G	C	*BjuOA09G47290*
A09	46,799,861	T	C	missense_variant	46,799,861	T	C	*BjuOA09G47290*
A09	46,799,897	C	T	missense_variant	46,799,897	C	T	*BjuOA09G47290*
A09	46,799,987	C	A	missense_variant	46,799,987	C	A	*BjuOA09G47290*
A09	46,800,041	G	T	missense_variant	-	-	-	*BjuOA09G47290*
A09	46,800,056	C	T	stop_gained	-	-	-	*BjuOA09G47290*
A09	46,800,095	G	C	missense_variant	46,800,095	G	C	*BjuOA09G47290*
A09	46,800,143	C	T	missense_variant	46,800,143	C	T	*BjuOA09G47290*
A09	46,800,194	T	C	missense_variant	-	-	-	*BjuOA09G47290*
A09	46,800,197	C	T	missense_variant	-	-	-	*BjuOA09G47290*
A09	46,800,242	T	C	missense_variant	46,800,242	T	C	*BjuOA09G47290*
A09	-	-	-	-	46,800,326	T	C	*BjuOA09G47290*
A09	46,800,374	A	G	missense_variant	46,800,374	A	G	*BjuOA09G47290*
A09	46,800,401	G	A	missense_variant	46,800,401	G	A	*BjuOA09G47290*
A09	-	-	-	-	46,800,511	A	G	*BjuOA09G47290*
A09	-	-	-	-	46,800,762	C	G	*BjuOA09G47290*
A09	46,801,226	A	G	synonymous_variant	46,801,226	A	G	*BjuOA09G47290*
A09	46,801,270	C	T	missense_variant	-	-	-	*BjuOA09G47290*
A09	46,801,286	G	A	5_prime_UTR_variant	-	-	-	*BjuOA09G47290*
A09	46,801,307	G	A	5_prime_UTR_variant	-	-	-	*BjuOA09G47290*
A09	46,801,337	A	T	5_prime_UTR_variant	-	-	-	*BjuOA09G47290*
A09	46,801,386	G	A	5_prime_UTR_variant	46,801,386	G	A	*BjuOA09G47290*
A09	46,801,436	A	G	5_prime_UTR_variant	46,801,436	A	G	*BjuOA09G47290*
A09	45,670,196	C	G	synonymous_var	-	-	-	*BjuOA09G45170*
A09	-	-	-	-	45,670,220	A	G	*BjuOA09G45170*
A09	45,670,292	T	C	synonymous_var	-	-	-	*BjuOA09G45170*
A09	45,670,968	A	G	synonymous_varian	-	-	-	*BjuOA09G45170*
A09	45,671,218	T	G	missense_variant	-	-	-	*BjuOA09G45170*
A09	45,671,381	G	T	synonymous	-	-	-	*BjuOA09G45170*
A09	45,671,597	G	C	synonymous_var	-	-	-	*BjuOA09G45170*

## Data Availability

The datasets generated during the current study are available in the Sequence Read Archive (SRA) database of National Center for Biotechnology Information (NCBI) under accession PRJNA1044267.

## Supplementary Materials

The following supporting information can be downloaded at: https://www.mdpi.com/article/10.3390/ijms25031573/s1.
